# Effect of Diabetes on Survival after Resection of Pancreatic Adenocarcinoma. A Prospective, Observational Study

**DOI:** 10.1371/journal.pone.0166008

**Published:** 2016-11-04

**Authors:** Gianpaolo Balzano, Erica Dugnani, Alessandra Gandolfi, Marina Scavini, Valentina Pasquale, Francesca Aleotti, Daniela Liberati, Gaetano Di Terlizzi, Giovanna Petrella, Michele Reni, Claudio Doglioni, Emanuele Bosi, Massimo Falconi, Lorenzo Piemonti

**Affiliations:** 1 Pancreatic Surgery Unit, Pancreas Translational & Clinical Research Center; IRCCS San Raffaele Scientific Institute, Milan, Italy; 2 San Raffaele Diabetes Research Institute (SR-DRI), IRCCS San Raffaele Scientific Institute, Milan, Italy; 3 Department of Medical Oncology, IRCCS San Raffaele Scientific Institute, Milan, Italy; 4 Department of Pathology, IRCCS San Raffaele Scientific Institute, Milan, Italy; 5 Vita-Salute San Raffaele University, Milan, Italy; University of Nebraska Medical Center, UNITED STATES

## Abstract

**Aim:**

To investigate the effect of diabetes mellitus (DM) on disease-free and overall post-resection survival of patients with pancreatic ductal adenocarcinoma (PDAC)

**Methods:**

Prospective observational study on patients admitted for pancreatic disease from January 2008 to October 2012. DM was classified as recent-onset (<48 months before PDAC diagnosis), longstanding (≥48 months before PDAC) or new onset (after surgery).

**Results:**

Of 296 patients, 140 had a diagnosis of DM prior to surgery (26 longstanding, 99 recent-onset, 15 with unknown duration). Median follow-up time was 5.4 ± 0.22 years. Patients with recent onset DM had poorer postoperative survival than patients without DM: disease-free survival and overall survival were 1.14±0.13 years and 1.52±0.12 years in recent onset DM, versus 1.3±0.15 years and 1.87±0.15 years in non-diabetic patients (p = 0.013 and p = 0.025, respectively). Longstanding DM and postoperative new onset DM had no impact on prognosis. Compared to cases without DM, patients with recent onset DM were more likely to have residual disease after surgery and to develop liver metastases during follow-up. Multivariate analysis confirmed recent onset DM was independently associated with PDAC relapse (hazard ratio 1.45 [1.06–1.99]).

**Conclusion:**

Preoperative recent onset DM has an impact on survival after the resection of PDAC.

## Introduction

The association between pancreatic ductal carcinoma (PDAC) and diabetes mellitus (DM) is complex [1–3] and numerous epidemiological studies have examined this relationship [4–8]. Meta-analyses of cohort and case-control studies demonstrated an increased risk of PDAC among individuals with DM [9–14]. The excess risk has been shown to be greater (5–8 fold) for a recent diagnosis of DM (generally defined as DM diagnosed in the 2 to 4 years before cancer diagnosis) [15–17] and to decline with diabetes duration (about 1.5 fold 4 years after DM diagnosis) [10]. However, an increased risk for PDAC has been reported also among individuals who had DM for 10 or more years [7, 10], suggesting that DM may play a causal role in pancreatic carcinogenesis [18, 19]. However, despite the documented association between PDAC and DM, relatively little is known about the impact of DM diagnosed prior to PDAC surgical removal and even less about of DM diagnosed postoperatively on the prognosis of PDAC. Several studies have focused on this topic, but their results are contradictory [20–29], possibly because of the different criteria for the diagnosis of DM (including self-reported, review of medical records, or biochemical criteria), the heterogeneity of the patient groups and the retrospective design. We recently conducted a prospective observational study to describe the clinical features, risk factors and etiopathogenetic aspects of patients with DM associated with pancreas disease (T3cDM) [30, 31]. This cohort allows us to analyse a population with PDAC (among the 651 recruits 364 had a diagnosis of PDAC), with the advantage of having: (i) clinical and biochemical data collected according to a standard protocol (prospective design); (ii) diabetes diagnosis based on fasting glucose or glycosylated hemoglobin instead of proxy or self-reported information; (iii) information on insulin secretion and sensitivity and/or islet autoimmunity; and (iv) planned follow-up. The aim of the present study is to evaluate the impact of DM on disease-free and overall survival after resection with curative intent of PDAC. Given the different pathophysiology we evaluated the association of DM with patient survival in patients with PDAC separately for long-standing DM, recent-onset DM and post resection DM.

## Material and Methods

### Eligible patients

From January 2008 to December 2012, 787 adult incident cases of pancreatic disease candidate to pancreatic surgery were admitted to the Pancreatic Surgery Unit of the S. Raffaele Scientific Institute and 651 eligible patients accepted to participate (see [30]). Among eligible patients we selected those with a diagnosis of pancreatic ductal carcinoma submitted to pancreatectomy with radical intent (296 cases). All patients provided a written informed consent to participate to the study and the San Raffaele Ethics Committee approved the study.

### Demographic, clinical data and medical history

At the entry into the study both inpatient and outpatient medical records of each participant were reviewed to abstract the following variables: gender, age, height, weight and BMI [both actual and 12 month before surgery (defined as the usual BMI)], family history of diabetes in first-degree relatives, date of diagnosis of PDAC and presenting sign/symptoms, concomitant diseases (classified as ever/never: non-pancreatic tumour, dyslipidaemia, hypertension, coronary artery disease, hepatic diseases, autoimmune diseases, thyroid dysfunction). In patients with previous diagnosis of DM the medical records were also reviewed to compute disease duration and diabetes medication. All abstracted data were validated with either an interview with the patient and/or a relative. Perioperative and pathologic data were also collected in a prospective electronic pancreatic surgery database.

### Blood biochemistry during hospitalization

Fasting blood samples were obtained during the preoperative work-up [upon or just before hospital admission, median 6 days (1–13) before surgery] and prior to hospital discharge [median 9 (7–12) days after surgery]. Baseline hemoglobin A_1c_ (HbA_1c_; Bio-Rad Variant II HbA_1C_ analyzer; Bio-Rad Laboratories, Munich, Germany), baseline and discharge fasting plasma glucose (FPG; glucose-oxidase method, Advia 2400; Siemens Diagnostics, Deerfield, IL) and baseline and discharge serum creatinine (kinetic alkaline picrate method, Advia 2400) were measured in all patients. Serum insulin (AIA-PACK IRI; Tosoh, Tokyo, Japan) was measured upon admission and prior to hospital discharge in 291 and 122 out of 364 patients, respectively. C-peptide (AIA-PACK C-Peptide; Tosoh) was measured at baseline and prior to hospital discharge in 135 and 67 out of 364 patients, respectively. Autoantibodies to glutamic acid decarboxylase (GADA), insulinoma-associated protein 2 (IA-2A), insulin (IAA) and zinc transporter 8 antigen (ZnT8A) were measured in 300 out of 364 patients by radio binding and immunoprecipitation assays as previously described [32]. Insulin resistance and β-cell function were estimated using the HOMA2 model (available from www.OCDEM.ox.ac.uk), following the recommendations for its appropriate use [33]. Estimation of glomerular filtration rate (eGFR) was calculated using the simplified modification of diet in renal disease (MDRD) formula [34]. To asses inflammatory biomarkers we calculated peripheral neutrophil-to-lymphocyte ratio (NLR) [35] and platelet-to-lymphocyte ratio (PLR), both reported as poor prognostic indicators in several malignancies, and lymphocyte-to-monocyte ratio (LMR) previously associated with a favorable prognosis for certain hematologic and solid tumors [36]. Preoperative nutritional status was evaluated in all study participants using Onodera’s prognostic nutrition index (PNI) calculated as 10 x albumin (g/dl) + 0.005 x total lymphocyte count (per mm^3^). A PNI value of at least 50 was defined as normal nutritional status, between 50 and 46 was regarded as mild malnutrition, between 45 and 40 as moderate malnutrition and less than 40 as severe malnutrition [37]. To grade nutritional status we computed the Geriatric Nutritional Risk Index (GNRI) [1.519 x albumin (g/L) + 41,7 x (weight / ideal weight)] [38]. A GNRI value of at least 98 was defined as normal, less than 98 was regarded as mild malnutrition, between 92 and 82 as moderate malnutrition and less than 82 as severe malnutrition.

### Diabetes definition and its duration

Study participants were defined as having diabetes if at least one fasting plasma glucose (FPG) was ≥7.0 mmol/l or HbA_1C_ was ≥ 6.5% (48 mmol/mol) or they were prescribed diabetes medications. At the time of PDAC diagnosis participants were classified as having: a) long-standing diabetes if they had a documented diagnosis of DM for ≥48 months; b) short-standing diabetes if they had a documented diagnosis of DM for <48 months [48-months is a period of time during which a PDAC is likely to become manifest (16)]; c) previously undiagnosed diabetes if participants were diagnosed with DM at the time of the diagnosis of PDAC; d) DM of uncertain duration if participants had a documented diagnosis of DM prior to PDAC diagnosis but duration was unknown. We define recent onset DM as the combination of short-standing and previously unknown DM. During follow up participants were classified as having new-onset DM if a documented diagnosis of DM was made after pancreatic surgery.

### Follow-up

Outpatient’s visits were scheduled one month after hospital discharge and every six months thereafter. Follow-up data were collected for all patients by reviewing electronic medical records and/or telephone interviews. Adjuvant chemo- or radiotherapy was prescribed when indicated and CT scan and blood tumour markers were performed every three to six months, based on the risk of recurrence. Medical history, including diagnosis of diabetes, disease-free survival and overall survival were recorded.

### Statistical Analysis

Data are presented as mean ± standard deviation (SD) or median and (interquartile range, IQR), according to their distribution. Variables with a normal distribution were compared with one-way unpaired or paired Student’s *t* test (two groups) or one-way ANOVA test (three or more groups). Variables with a non-normal distribution were compared with Wilcoxon signed-rank test, or Mann-Whitney U test (two groups), or Kruskal-Wallis test (three or more groups). Categorical variables were compared with the chi-square test or Fisher’s exact test, as appropriate. Disease-free and overall survival was estimated according to Kaplan-Meier. Association between variables were evaluated by logistic regression and Cox regression analysis as appropriate. The multivariate analysis was performed using variables significant in the univariate analysis at the level of 0.20. All statistical analyses were performed using the SPSS statistical software, version 13.0 (SPSS Inc, Chicago, Illinois, USA).

## Results

### Study participants

When PDAC was diagnosed, 140 out of 296 patients (47.3%) already had DM. Long-duration DM, short-duration DM and previously undiagnosed DM accounted for 18.6% (n = 26), 40% (n = 56) and 30.7% (n = 43) of the patients with DM, respectively, while 10.7% (n = 15) of the patients had a DM of uncertain duration. Patients with DM of uncertain duration (n = 15) were excluded from subsequent analysis. Of 281 cases in our series, 173 (61.6%) underwent pancreaticoduodenectomy, 72 (25.6%) distal pancreatectomy and 36 (12.8%) total pancreatectomy. As of December 2015, median follow-up time after diagnosis was 5.4 ± 0.22 (standard error) years. Two-hundred and sixteen patients died during follow-up (76.9%). All the 65 patients known to be alive at the end of the observation period were censored at the latest follow-up visit, with a median follow-up time of 5.1 ± 0.29 years.

### Effect of pre-operative DM on PDAC outcome

The general characteristics of the study participants are reported in Table 1. Patients with recent onset DM were older than patients without DM (69.1±9 vs 65.3±11 years, p<0.05), had higher FPG and HbA_1C_ at admission and higher prevalence of family history of diabetes. The median duration of recent-onset diabetes was 0.77±1.1 years prior to PDAC diagnosis (Table 1). Both beta-cell dysfunction and insulin resistance contributed to the pathogenesis of recent onset diabetes: with respect to patients without DM, C-pep HOMA2-%B was 71 (52–99) vs 121 (89–155; p<0.05) and insulin HOMA2-%S was 74 (43–133) vs 102 (75–160; p<0.05), respectively (Table 1). As for the treatment of diabetes 23.2% of patients were treated with insulin, 25.3% with oral diabetes medications, 3% with both insulin and oral diabetes medications, and 8.1% with lifestyle modifications. Seventeen percent of patients with recent-onset DM tested positive for at least one autoantibody and 1% for more than one: IAA was the most frequent specificity (12%) followed by GADA (3%), IA-2A (2%) and ZnT8A (1%). Also long-lasting DM patients were older than patients without DM (69.6±7.6 years, p<0.05), had higher FPG and HbA_1C_ at admission and higher prevalence of family history of diabetes. The age at onset of long-lasting diabetes was 54±9.9 years, with an overall median DM duration of 15±9.9 years at PDAC diagnosis (Table 1). Similarly to recent-onset diabetes, both beta-cell dysfunction and insulin resistance contributed to the pathogenesis of this diabetes: C-pep HOMA2-%B was 61 (25–131) vs 121 (89–155; p<0.05) and insulin HOMA2-%S was 60 (24–117) vs 102 (75–160; p<0.05) in long-standing diabetes and patients without DM, respectively (Table 1). As for the treatment of diabetes 61.5% of the patients were treated with insulin, 38.5% with oral diabetes medications, and 11.5% with both insulin and oral diabetes medications. Twenty-nine percent of the patients with long-standing DM tested positive for at least one autoantibody and 4% for more than one: IAA was the most frequent specificity (29%), followed by GADA (8%). Cardiovascular comorbidity, hyperlipidaemia and prostatic hypertrophy were all associated with long-standing DM.

**Table 1:** Patient characteristics by types of preoperative diabetes.

	No diabetes	Recent onset diabetes	Long-standing diabetes	p
N	156	99	26	
Sex (F/M)	82/74	46/53	9/17	0.202
Age at admission	65.3±11	69.1±9 ^a^	69.6±7.6 ^a^	0.007
Age at diabetes onset	-	68.3±9.2	54±9.9	<0.001
Diabetes duration (years)	-	0.77±1.1	15±9.9	<0.001
Diabetes familiarity	21%	38% ^a^	64% ^a^^,^^b^	<0.001
FPG (mg/dl)				
- Admission	94±14	146±48 ^a^	154±76 ^a^	<0.001
- Discharge	115±33	142±45 ^a^	170±51 ^a^^,^^b^	<0.001
- Last	119±40	148±57 ^a^	148±53 ^a^^,^^b^	<0.001
HbA_1__c_ [%(mmol/mol)]				
- Admission	5.56±0.57	6.9±1.3 ^a^	8.22±1.65 ^a^^,^^b^	<0.001
- 1M after discharge	5.73±0.78	6.93±1.4 ^a^	8±1.44 ^a^^,^^b^	<0.001
Fasting insulin (pmol/l)	44 (29–60)	51 (31–109)	62 (37–138) ^a^	0.021
Fasting C-peptide (nmol/l)	0.63 (0.53–0.88)	0.76 (0.44–1.26)	0.43^a^^,^^b^ (0.19–0.60)	0.05
Insulin HOMA2-%B				
- Admission	86 (60–121)	53 (33–78) ^a^	63 (40–115)	<0.001
- Discharge	62 (45–99)	35 (27–53)	27 (17–49)	<0.001
C-pep HOMA2-%B				
- Admission	121 (89–155)	71 (52–99) ^a^	61 (25–131) ^a^	<0.001
- Discharge	80 (54–111)	59 (34–79) ^a^	17 (12–25) ^a^^,^^b^	<0.001
Insulin HOMA2-%S				
- Admission	102 (75–160)	74 (43–133) ^a^	60 (24–117) ^a^	<0.001
- Discharge	102 (57–180)	113 (78–228)	92 (40–163)	0.41
Therapy				
- Insulin	-	23.2%	61.5%	<0.001
- Insulin only	-	20.2%	50%	
- Insulin+oral medication	-	3%	11.5%	
- Oral medication only	-	25.3%	38.5%	
- Lifestyle modifications	-	8.1%	0%	
- No therapy	-	43.4%	0%	
Autoimmunity				
- 0Ab	90%	83%	67%	0.048
- 1Ab	9.2%	16%	29%	
- >1Ab	0.8%	1%	4%	
- GADA+	4%	3%	8%	0.56
- IA-2A+	1%	2%	0%	0.71
- IAA+	5%	12% ^a^	29% ^a^	<0.001
- ZnT8A+	2%	1%	0%	0.82
Weight (Kg)	68.6±14	68±13	74±15	0.17
BMI	24.3±4.1	24.5±4.6	26.1±5.7	0.19
Last year weight loss (Kg)	4 (8–0)	6 (10–3) ^a^	5.5 (8.7–0.5)	0.012
Creatinine (mcmol/l)	67.4±19	71.2±21.6	110±164 ^a^^,^^b^	0.001
eGFR (mL/min/1.73 m^2^)	101±31	96±27	89±33	0.105
Onodera index (PNI)	48 (43–50)	47 (43–50)	45 (43–49)	0.65
Geriatric Nutritional Risk Index (GNRI)	104 (97–111)	101 (95–110)	105 (98–114)	0.29
Neutrophil-to-lymphocyte ratio (NLR)	2.6 (1.7–3.7)	2.4 (1.7–4)	2 (1.4–3)	0.26
Lymphocyte-to-monocyte ratio (LMR)	3 (2–3.9)	3 (2.2–4)	3.4 (1.8–5)	0.58
Platelet-to-lymphocyte ratio (PLR)	149 (119–213)	133 (88–191)	105 (76–184) ^a^	0.046
Commorbidities:				
- Cardiovascular disease	16.4%	25.7%	59.1% ^a^^,^^b^	<0.001
- Hypertension	53%	53%	68%	0.4
- Hyperlipidemia	13%	17%	36% ^a^^,^^b^	0.029
- Hepatopathy	5%	8%	0%	0.29
- Neoplastic disease	18%	25%	7%	0.33
- Prostatic hypertrophy	6%	12%	23% ^a^	0.031
- Dysthyroidism	15%	4% ^a^	4% ^a^	0.026
- Autoimmune disease	7%	3%	4%	0.44

^a^ p<0.05 vs no diabetes

^b^ p<0.05 vs recent onset diabetes

^c^ p<0.05 vs long lasting diabetes at post hoc analysis.

**Fig 1:**
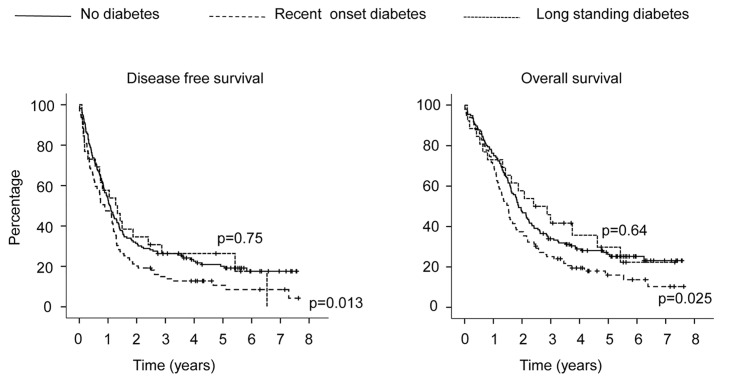
Effect of pre-operative DM on PDAC outcome. Kaplan-Meier estimates of disease-free (left) and overall (right) survival stratified according to pre-operative DM for 281 patients with a diagnosis of pancreatic ductal carcinoma submitted to pancreatectomy with radical intent from the Pancreatic Surgery Unit of the S. Raffaele Scientific Institute (2008–2012). At the time of PDAC diagnosis participants were classified as having: long-standing diabetes if they had a documented diagnosis of DM for ≥48 months; recent onset DM if participants were diagnosed with DM at the time of the diagnosis of PDAC or had a documented diagnosis of DM for <48 months. The *X-axis* shows the time since pancreatectomy. P value of *log*-*rank test* vs no diabetes are reported.

**Fig 2:**
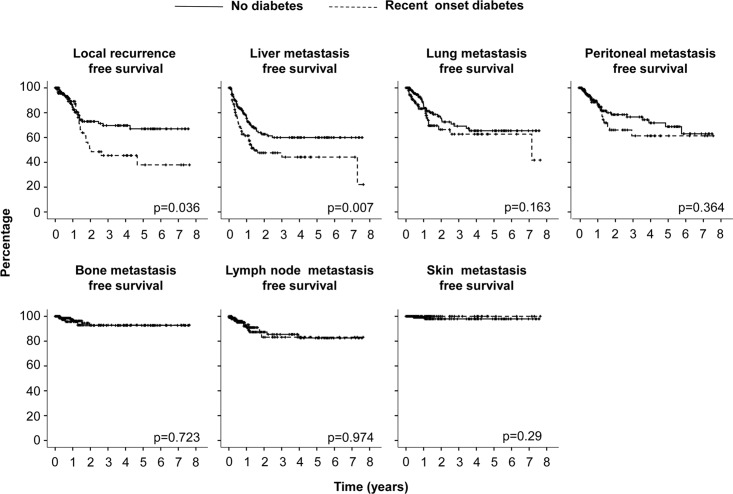
Effect of pre-operative recent onset DM on PDAC relapse. Kaplan-Meier estimates of disease free survival and stratified according to seven main recurrence sites for 255 patients (156 no diabetes; 99 recent onset diabetes) with a diagnosis of pancreatic ductal carcinoma submitted to pancreatectomy with radical intent from the Pancreatic Surgery Unit of the S. Raffaele Scientific Institute (2008–2012). The *X-axis* shows the time since pancreatectomy and the *y-axis* the PDAC recurrence-free probability. P value of *log*-*rank test* vs no diabetes are reported.

Based on preoperative glycaemic status, disease free-survival and overall survival were analysed (Fig 1). Among 156 patients without DM at the time of PDAC diagnosis (median follow-up 5.4±0.19 years) relapse was detected in 98 patients after surgery (62.8%) and 115 (73.7%) died: the median disease-free survival was 477±57 days and overall survival 684±54 days. Among 99 patients with recent-onset DM (median follow-up 5±0.63 years) relapse was detected in 74 patients after surgery (74.7%; p = 0.048 vs patients without DM) and 99 died (83.8%; 0.059 vs patients without DM): the median disease-free survival was 417±49 days (p = 0.013 vs patients without DM) and overall survival 555±43 days (p = 0.025 vs patients without DM). Among 26 patients with long-lasting DM (median follow-up 5.5±1.06 years) relapse occurred in 15 patients after surgery (57.7%; 0.62 vs patients without DM) and 18 died (69.2%; 0.63 vs patients without DM): the median disease-free survival was 519±64 days (p = 0.75 vs patients without DM) and overall survival 876±249 days (p = 0.64 vs patients without DM). Tumor-related characteristics are reported in S1 Table. Compared to cases without DM, patients with recent-onset DM were more likely to have residual disease after the surgery and to develop liver metastases during the follow-up, while tumour location, stage, grading, therapy were not different. Concordantly recent-onset diabetes had a significant impact on liver metastasis free-survival and local recurrence-free survival (Fig 2). Taken together these data suggest that preoperative recent-onset diabetes, but not long-lasting DM, has an impact on the prognosis of PDAC.

**Fig 3:**
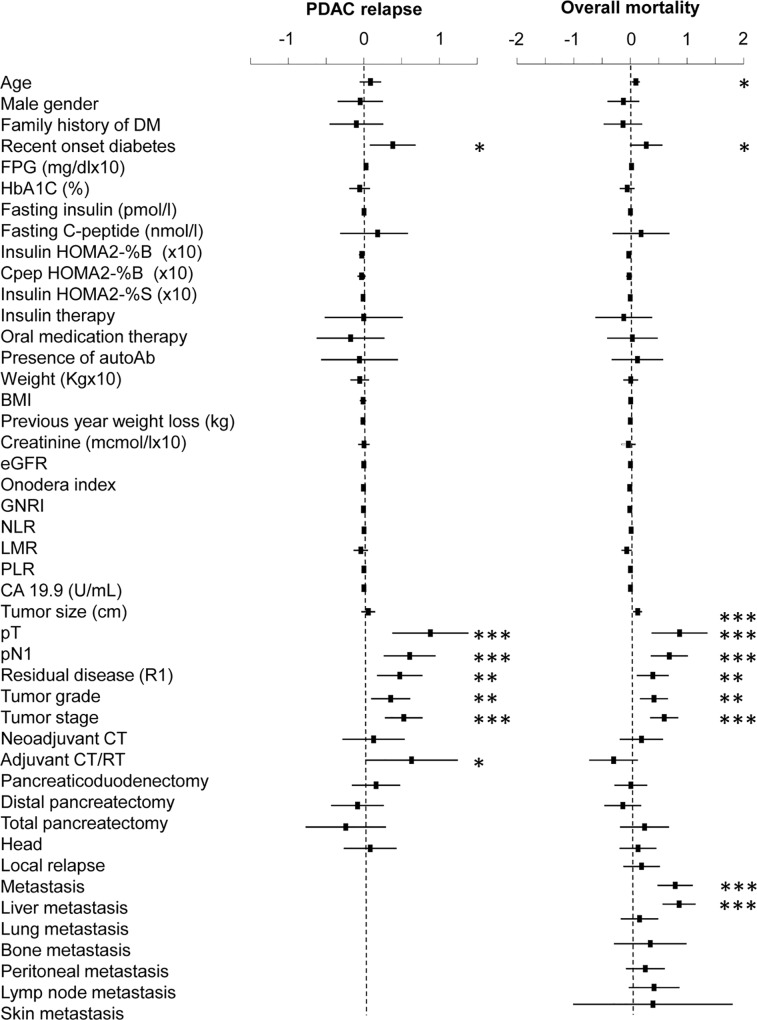
Univariate hazard ratios for PDAC relapse and mortality. The association between baseline variables and PDAC relapse or mortality was assessed by a Cox regression analysis including patients with recent-onset DM and without DM. All analyzed variables are presented. Dots represent the Hazard Ratio (HR) after natural log transformation, lines the 95% confidence intervals. *p<0.05; **p<0.01; ***p<0.001. FPG = Fasting Plasma Glucose; HOMA = Homeostatic Model Assessment; GNRI = Geriatric Nutritional Risk Index; NLR = neutrophil-to-lymphocyte ratio; LMR = lymphocyte-to-monocyte ratio; PLR = platelet-to-lymphocyte ratio.

To better characterized factors associated with disease-free and overall survival, a Cox regression analysis was performed including patients with recent-onset DM and patients without diabetes (n = 255). In the univariate analysis (Fig 3) we found an association between disease relapse and recent-onset diabetes [HR 1.46 (1.08–1.98); p = 0.013], pT [HR = 2.41 (1.4–3.99); p<0.001], pN1 [HR = 1.83 (1.3–2.58); p<0.001], microscopic residual disease [HR = 1.61 (1.19–2.17); p = 0.002], tumor grade [HR = 1.42 (1.1–1.84); p = 0.007], tumor stage [HR = 1.69 (1.32–2.17); p<0.001], adjuvant CT/RT [HR = 1.88 (1.019–3.46); p = 0.043]. The multivariate analysis (S2 Table) confirmed tumour stage and recent onset diabetes as independent predictors of PDAC relapse. Concordantly, recent onset diabetes [HR = 1.32 (0.99–1.76); p = 0.05], tumor size [HR = 1.13 (1.05–1.22); p<0.001] pT [HR = 2.38 (1.46–3.89); p<0.001], pN1 [HR = 1.98 (1.4–2.7); p<0.001], microscopic residual disease [HR = 1.48 (1.12–1.96); p = 0.006], tumor grade [HR = 1.51 (1.2–1.9); p<0.001], tumor stage [HR = 1.81 (1.41–2.32) p<0.001] and the development of liver metastasis [HR = 2.36 (1.76–3.15); p<0.001] were associated with lower survival in the univariate analysis after adjusting for age and sex (Fig 3). The multivariate analysis (S2 Table) confirmed tumour stage, tumour size and development of liver metastasis as independent predictors of PDAC mortality.

### Effect of post-operative DM on PDAC outcome

**Fig 4:**
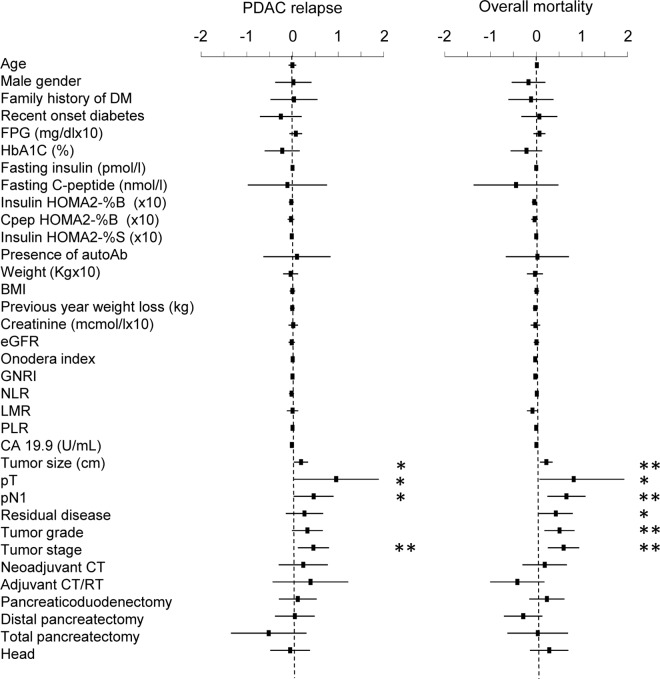
Univariate hazard ratios for PDAC relapse and mortality. The association between baseline variables and PDAC relapse or mortality was assessed by Cox regression analysis with time-dependent covariates including patients without DM at PDAC diagnosis. All analyzed variables are presented. Dots represent the Hazard Ratio after natural log transformation, lines the 95% confidence intervals. *p<0.05; **p<0.01; ***p<0.001. FPG = Fasting Plasma Glucose; HOMA = Homeostatic Model Assessment; GNRI = Geriatric Nutritional Risk Index; NLR = neutrophil-to-lymphocyte ratio; LMR = lymphocyte-to-monocyte ratio; PLR = platelet-to-lymphocyte ratio.

Among the 156 patients without DM at the time of PDAC diagnosis, 60 (20.3% of total) developed DM after pancreatic resection, while 96 (32.4%) were without DM at the time of PDAC diagnosis and did not develop it during follow-up. The general characteristics of study participants are reported in Table 2. The age at onset of patients with new-onset diabetes was 66.1±10.2 years, with a diabetes-free median time of 30±48 days after discharge. As for the treatment of diabetes, at last follow-up 38.3% of the patients were treated with insulin, 15% with oral diabetes medications. Factors associated with postoperative new onset DM in the univariate Cox regression analysis were: family history of DM, distal pancreatectomy, pre-operative higher weight, BMI and FPG; all these factors, with the exception of weight and BMI, were confirmed in the multivariate analysis (S1 Fig). To characterize the association between new onset DM and disease-free or overall survival, a Cox regression with time dependent covariates was performed. Tumor size, pT, pN and tumor stage were associated with both PDAC relapse and overall mortality in the univariate analysis (Fig 4). Residual disease and tumor grade were associated with overall mortality. Multivariate analysis (S2 Table) confirmed tumor stage [HR = 1.44 (1.01–2.06); p = 0.042] as a variable independently associated with PDAC relapse. New onset diabetes was not associated with PDAC relapse or mortality.

**Table 2:** Patients characteristics by postoperative diabetic status.

	No diabetes	New onset diabetes	
N	96	60	
Sex (F/M)	53/43	29/31	0.40
Age at admission	65±12	65.6±10	0.78
Age at diabetes onset	-	66.11±10.2	
Diabetes familiarity	11%	37%	<0.001
FPG (mg/dl)			
- Admission	91±9	99±12	0.003
- Discharge	104±19	133±43	<0.001
- Last	100±15	149±49	<0.001
HbA_1C_ (%)			
- Admission	5.50±0.56	5.65±0.56	0.152
- 1M after discharge	5.53±0.44	6±1	0.031
- Last	5.78±0.59	7.2±0.9	0.004
Fasting insulin (pmol/l)	42 (28–60)	49 (35–69)	0.26
Fasting C-peptide (nmol/l)	0.64 (0.51–0.80)	0.61 (0.53–1.1)	0.82
Insulin HOMA2-%B	84(62–126)	87 (61–119)	0.46
C-pep HOMA2-%B	121 (98–145)	121 (98–145)	0.59
Insulin HOMA2-%S	108 (80–167)	94 (68–144)	0.21
Therapy			
- Insulin	-	38.3%	<0.001
- Oral medication only	-	15%	
- Lifestyle modifications	-	3.3%	
- No therapy	-	43.4%	
Autoimmunity			
- 0Ab	90%	90%	0.42
- 1Ab	10%	8%	
- >1Ab	0%	2%	
- GADA+	4%	4%	0.94
- IA-2A+	2%	0%	0.26
- IAA+	3%	8%	0.25
- ZnT8A+	1%	2%	0.75
Weight (Kg)	66.9±13	71±22	0.075
BMI	23.7±4	25.4±4.2	0.021
Last year weight loss (kg)	3 (8–0)	4.5 (8–0)	0.87
Creatinine (mcmol/l)	66.8±19	68.3±20.1	0.66
eGFR (mL/min/1.73 m^2^)	100±28	103±36	0.63
Onodera index (PNI)	48 (44–51)	48 (44–51)	0.36
Geriatric Nutritional Risk Index (GNRI)	103 (96–107)	107 (97–113)	0.091
Neutrophil-to-lymphocyte ratio (NLR)	2.6 (1.7–3.6)	2.4 (1.7–3.3)	0.13
Lymphocyte-to-monocyte ratio (LMR)	3 (1.8–3.9)	3.2 (2.4–4.5)	0.34
Platelet-to-lymphocyte ratio (PLR)	159 (110–232)	125 (96–165)	0.009
Comorbidities:			
- Cardiovascular disease	15%	18%	0.69
- Hypertension	53%	53%	0.97
- Hyperlipidemia	17%	8%	0.16
- Hepatopathy	3%	8%	0.18
- Neoplastic disease	14%	24%	0.15
- Prostatic hypertrophy	8%	2%	0.14
- Dysthyroidism	15%	15%	0.92
- Autoimmune disease	7%	7%	0.94

## Discussion

In the last years several cohort studies have showed that cancer patients with preexisting DM are generally at increased risk of adverse long-term outcomes compared to patients without DM [11, 39, 40]. In the field of PDAC, the prognostic relevance of preoperative DM is still controversial. In retrospective studies DM was associated with reduced survival in patients undergoing resection for PDAC [21, 26, 29, 41–45]; on the other hand, DM had no impact on overall survival in other studies [46–50] or was unexpectedly associated to a reduced risk of death [51–53]. Inconsistent findings were also reported when DM was stratified by duration of diabetes: recent-onset DM, but not long-standing DM was associated with reduced PDAC survival in some studies [10, 29], while others did not find this association [23, 24, 43, 54, 55]. The inconsistency of those observations is likely due to differences in DM definition, small sample size, study populations with different stages of disease and the retrospective design, with no controlling for potential confounders. Consequently there is a need for further investigation of the relationship between DM and prognosis of PDAC [56]. Taking advantage of a prospective observational study conducted to describe T3cDM [30], we evaluated the impact of DM on disease-free survival, recurrence pattern and overall survival after potentially curative resection of PDAC limiting the effect of the potential confounders which may have biased previous studies. Taken together our data suggest that pre-operative recent-onset DM, but not long-lasting DM or post-operative new-onset DM, has an impact on the prognosis of PDAC. Our results are in agreement with a recent meta-analysis reporting that preexisting DM in PDAC is associated with increased risk of mortality [57]. Of note, the meta-analysis showed that the risk of mortality was higher in patients with resected or resectable cancer than in those with nonresectable cancer and was higher in patients with recent-onset DM than in those with long-lasting DM [57].

It remains unclear the possible cause of the relation between recent onset DM and prognosis of PDAC. Since long-standing DM and postoperative new onset DM did not affect survival, the poorer prognosis of patients with recent onset DM does not seem related to hyperglycemia, nor to insulin resistance and hyperinsulinemia, nor to poor overall health that may render patients more prone to complications following surgical and adjuvant therapies. In fact glucose exposure, both in terms of FPG and HbA_1c_, was higher in long-standing than in recent-onset DM, either before or after PDAC removal. Similarly, during follow-up FPG and HbA_1C_ were as expected higher in new-onset DM than in patients without DM. Hyperinsulinemia and low insulin sensitivity were both present in recent-onset and, even more prevalent, in long-standing DM. Mortality within 90 day from surgery in the different groups were similar, and did not significantly contribute to the observed differences in long-term survival. Moreover, long-standing DM showed the strongest association with cardiovascular comorbidity and dyslipidemia. Taken together these data suggest that the association of DM with survival of PDAC patients is more complex than previously hypothesized. There are several potential explanations for our results. We can speculate that the loss of pancreatic parenchyma leading to the DM recent onset may be the consequence of a more aggressive cancer or of a tumour with longer duration before diagnosis. This hypothesis could be supported by the presence of a R1 resection rate higher in recent onset diabetes than in the other two groups, a difference that could be responsible for the worse behavior. To address this issue we evaluated the disease-free survival and overall survival for each of the 3 groups for patients with R0 resection alone and R1 resections alone (data not shown). The recent onset diabetes showed a clear trend for less median overall survival and disease free survival independently by the R1 status, speaking against this hypothesis.

Another possibility could be that under recent-onset hyperglycemic conditions, an increased level of oxidative stress and proinflammatory factors cause pancreatic nerve damage and an inflammatory response [58], which simultaneously facilitates cancer cell proliferation, migration, and metastasis [59]. Testing these postulated explanations is beyond the scope of this study, but their exploration should be continued in future research.

As previously reported [30] our cohort of patients presents some biases: patients studied were admitted for surgery (potential selection bias) in a tertiary-care academic center (potential referral bias). Moreover, in about 10% of patients with DM, it was not possible to document disease duration. Our use of 48 months as the cutoff for classification of recent-onset versus long-standing DM is questionable. However, the utilization of this threshold is based on studies that demonstrate progressive increase in prevalence of DM within 5 years prior to index date of cancer diagnosis [15]. We also analyzed our data using 24 months as an alternative cutoff for recent-onset DM (data not shown). In this scenario, 16 patients moved from the recent-onset category to the long-standing group. Major trends in clinicopathologic profile and survival remained the same.

## Conclusion

In conclusion, our study has clearly shown that a recent diagnosis of DM preceding the diagnosis of PDAC is independently associated with survival after PDAC resection. Etiologies underlying this association are unclear, and require additional investigations.

## Supporting Information

S1 Fig Univariate and multivariate hazard ratios for post-operative new onset DM. The associations between patients’ characteristics and new onset diabetes were assessed using Cox regression. All analysed variables are presented. Dots represent the Hazard Ratio (HR) after natural log transformation, lines the 95% confidence intervals. *p<0.05; **p<0.01; ***p<0.001. FPG = Fasting Plasma Glucose; HOMA = Homeostatic Model Assessment; GNRI = Geriatric Nutritional Risk Index; NLR = neutrophil-to-lymphocyte ratio; LMR = lymphocyte-to-monocyte ratio; PLR = platelet-to-lymphocyte ratio.
(PPTX)

S1 Table Tumor-related characteristics by types of diabetes. (DOCX)

S2 Table Cox proportional hazard models of the predictors of PDAC relapse and Mortality by multivariate analysis. (DOCX)
