# Supplementary material for: Effect of Diabetes on Survival after Resection of Pancreatic Adenocarcinoma. A Prospective, Observational Study
Source: PLoS One. 2016 Nov 4;11(11):e0166008. doi: 10.1371/journal.pone.0166008 (PMC5096703; doi:10.1371/journal.pone.0166008)
Supplement: S1 Fig — The associations between patients’ characteristics and new onset diabetes were assessed using Cox regression. All analysed variables are presented. Dots represent the Hazard Ratio (HR) after natural log transformation, lines the 95% confidence intervals. *p<0.05; **p<0.01; ***p<0.001. FPG = Fasting Plasma Glucose; HOMA = Homeostatic Model Assessment; GNRI = Geriatric Nutritional Risk Index; NLR = neutrophil-to-lymphocyte ratio; LMR = lymphocyte-to-monocyte ratio; PLR = platelet-to-lymphocyte ratio. (PPTX) [file pone.0166008.s001.pptx]

## Slide 1
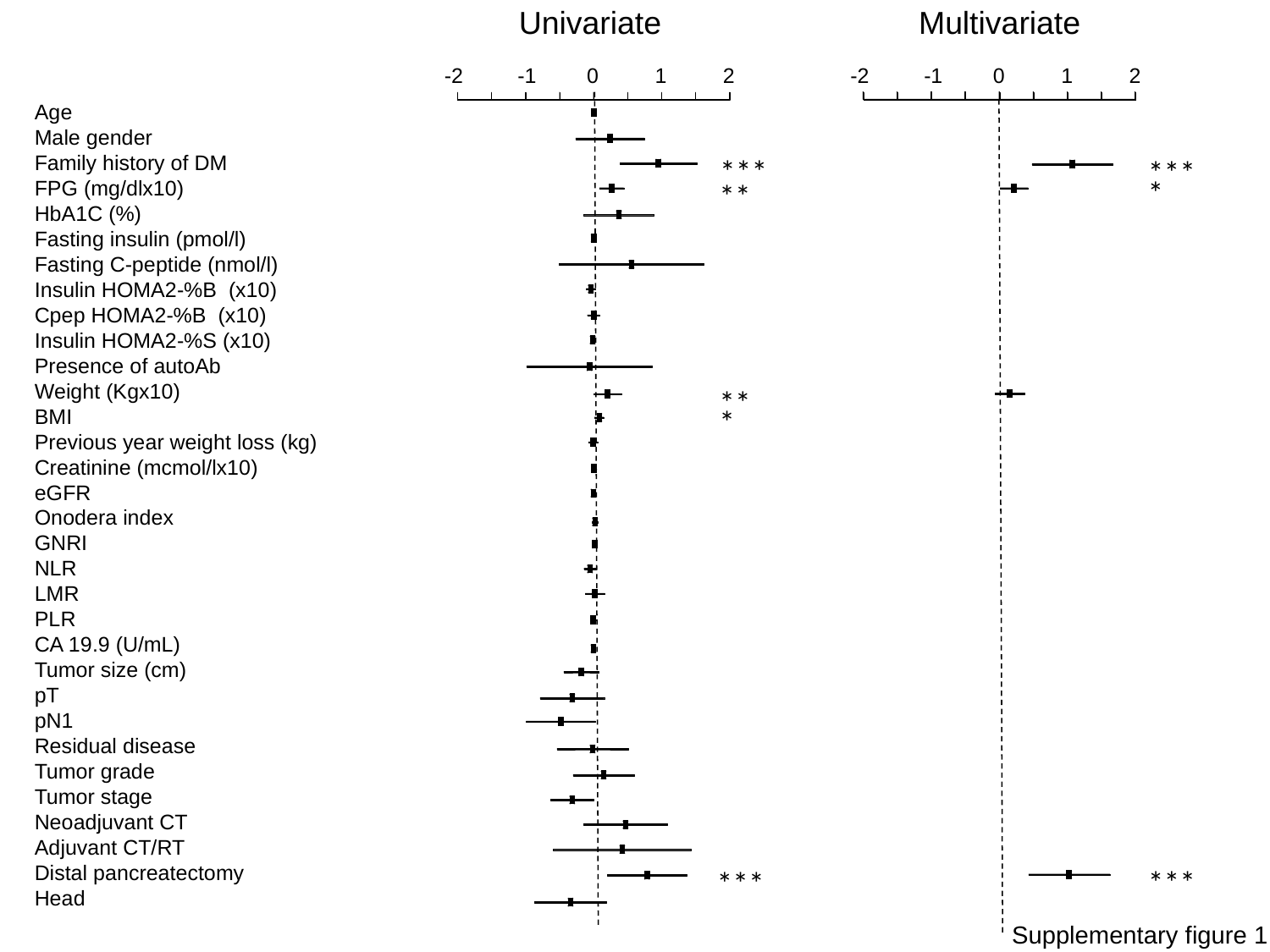

Univariate
Multivariate
-2
-1
0
1
2
-2
-1
0
1
2
Age
Male gender
Family history of DM
FPG (mg/dlx10)
HbA1C (%)
Fasting insulin (pmol/l)
Fasting C-peptide (nmol/l)
Insulin HOMA2-%B (x10)
Cpep HOMA2-%B (x10)
Insulin HOMA2-%S (x10)
Presence of autoAb
Weight (Kgx10)
BMI
Previous year weight loss (kg)
Creatinine (mcmol/lx10)
eGFR
Onodera index
GNRI
NLR
LMR
PLR
CA 19.9 (U/mL)
Tumor size (cm)
pT
pN1
Residual disease
Tumor grade
Tumor stage
Neoadjuvant CT
Adjuvant CT/RT
Distal pancreatectomy
Head
***
***
*
**
**
*
***
***
Supplementary figure 1
