# Supplementary material for: Effect of Diabetes on Survival after Resection of Pancreatic Adenocarcinoma. A Prospective, Observational Study
Source: PLoS One. 2016 Nov 4;11(11):e0166008. doi: 10.1371/journal.pone.0166008 (PMC5096703; doi:10.1371/journal.pone.0166008)
Supplement: S1 Table — (DOCX) [file pone.0166008.s002.docx]

**S1 Table. Tumor-related characteristics by types of diabetes**

|  | No diabetes | Recent onset diabetes | Long-standing diabetes | p |
| --- | --- | --- | --- | --- |
| N | 156 | 99 | 26 |  |
| CA 19.9 (U/mL) | 105 (27-239) | 111 (27-262) | 170 (52-287) | 0.43 |
| CA 15.3 (U/mL) | 21 (16-30) | 24 (17-32) | 19 (17-29.5) | 0.49 |
| Tumor size (cm) | 2.5 (2-3.2) | 2.7 (2-3.6) | 2.6 (2-3) | 0.35 |
| Tumor site head | 71.8% | 79.8% | 65.4% | 0.209 |
| pT:   - 1 - 2 - 3 - 4 | 3.2%  1.3%  94.9%  0.6% | 5.1%  5.1%  85.9%  4% | 3.8%  3.8%  92.3%  0% | 0.27 |
| pN1 | 67.3% | 73.7% | 53.8% | 0.14 |
| pM1 | 0% | 0% | 0% | 1 |
| R1 | 37.8% | 55.6% ^a,c^ | 38.5% | 0.017 |
| Tumor grade:   - G1 - G2 - G3 | 5.1%  57.1%  37.8% | 7.2%  53.6%  39.2% | 3.8%  53.8%  42.3% | 0.91 |
| Cancer stage:   - 1A - 1B - 2A - 2B - 3 | 3.2%  1.3%  28.2%  66.7%  0.6% | 5.1%  3%  18.2%  69.7%  4% | 3.8%  3.8%  38.5%  53.8%  0% | 0.19 |
| Therapy   - neoadjuvant CT - adjuvant CT/RT - pancreaticoduodenectomy - distal pancreatectomy - total pancreatectomy | 16.7%  89%  62.8%  28.2%  9% | 15.2%  82.1%  62.6%  20.2%  17.2% | 30.8%  88.5%  50%  30.8%  19.2% | 0.16  0.29  0.44  0.29  0.09 |
| Presenting sign/symptoms:   - Jaundice - Abdominal or back pain - Weight loss and poor appetite - Incidentaloma - Digestive problems - Cancer Markers - Astenia - Diabetes - Diarrhea - Fever | 42.4%  44.1%  14.7%  16.9%  13.6%  11.9%  5.1%  0%  0.8%  3.4% | 47.2%  29.2% ^a^  19.7%  12.5%  8.3%  8.3%  5.6%  9.7%^a^  6.9%  0% | 40%  20% ^a^  15%  25%  10%  10%  5%  10% ^a^  5%  5% | 0.75  0.031  0.652  0.384  0.53  0.74  0.99  0.002  0.069  0.239 |
| Death within 90 from surgery | 5.1% | 6.1% | 11.5% | 0.4 |
| Tumor relapse   - Local - Metastasis - Local+metastasis - None - Died w/o relapse | 7.1%  42.9%  12.8%  19.2%  17.9% | 11.1%  47.5%  16.2%  14.1%  11.1% | 11.5%  38.5%  7.7%  23.1%  19.2% | 0.56 |
| Metastasis   - Liver - Lung - Bone - Peritoneum - Lymph nodes - Skin | 30.1%  19.2%  3.8%  17.3%  9.7%  1.3% | 45.5%^a,c^  23.2%  4%  18.2%  8.1%  0% | 23.1%  26.9%  0%  0%  0%  0% | 0.018  0.57  0.59  0.064  0.25  0.44 |

^a^ p<0.05 vs no diabetes, ^b^ p<0.05 vs recent onset diabetes, ^c^ p<0.05 vs long lasting diabetes at post hoc analysis
