# Supplementary material for: Effect of Diabetes on Survival after Resection of Pancreatic Adenocarcinoma. A Prospective, Observational Study
Source: PLoS One. 2016 Nov 4;11(11):e0166008. doi: 10.1371/journal.pone.0166008 (PMC5096703; doi:10.1371/journal.pone.0166008)
Supplement: S2 Table — (DOCX) [file pone.0166008.s003.docx]

S2 Table. Cox proportional hazard models of the predictors of PDAC relapse and Mortality by multivariate analysis

| **Recent onset diabetes** |  | |  | |
| --- | --- | --- | --- | --- |
| **Adjusted for variables significant at p<0.05 in the univariate analysis** | **PDAC relapse** | | Overall mortality^a^ | |
|  | HR (95% CI) | p | HR (95% CI) | p |
| Recent onset diabetes | 1.45 (1.06-1.99) | 0.021 | 1.1 (0.8-1.51) | 0.53 |
| Tumor size | - | - | 1.1 (1.001-1.2) | 0.033 |
| Tumor stage | 1.49 (1.14-1.96) | 0.004 | 1.56 (1.19-2.05) | 0.001 |
| Tumor grade | 1.21 (0.91-1.6) | 0.19 | 1.27 (0.95-1.68) | 0.095 |
| Microscopic residual disease | 1.19 (0.86-1.64) | 0.29 | 0.99 (0.73-1.35) | 0.99 |
| Development of liver metastasis | - | - | 2.04 (1.5-2.7) | <0.001 |
| Adjuvant CT/RT | 1.15 (0.61-2.18) | 0.67 | - | - |
|  |  |  |  |  |
| **New onset diabetes** |  | |  | |
| **Adjusted for variables significant at p˂0.1 in the univariate analysis** | **PDAC relapse** | | Overall mortality^a^ | |
|  | HR (95% CI) | p | HR (95% CI) | p |
| New onset diabetes | 0.88 (0.55-1.41) | 0.6 | 0.89 (0.55-1.42) | 0.62 |
| Tumor size | 1.16 (0.99-1.37) | 0.061 | 1.16 (0.99-1.37) | 0.061 |
| Tumor stage | 1.44 (1.01-2.06) | 0.042 | 1.45 (0.99-2.11) | 0.053 |
| Tumor grade | 1.39 (0.96-1.99) | 0.078 | 1.4 (0.97-2.02) | 0.071 |
| Microscopic residual disease | - | - | 1.04 (0.66-1.6) | 0.87 |

^a^adjusted for age and sex
